# Conduit size and the risk of delayed gastric conduit emptying after esophagectomy

**DOI:** 10.1007/s00595-026-03395-w

**Published:** 2026-07-14

**Authors:** Yuta Sato, Yoshihiro Tanaka, Yuji Hatanaka, Seito Fujibayashi, Noriki Mitsui, Ryoma Yokoi, Takeshi Horaguchi, Keita Matsumoto, Masahiro Fukada, Itaru Yasufuku, Ryuichi Asai, Jesse Yu Tajima, Nobuhisa Matsuhashi

**Affiliations:** 1 Department of Gastroenterological Surgery and Pediatric Surgery, Gifu University Graduate School of Medicine, 1-1 Yanagido, Gifu City, 501-1194 Gifu Prefecture Japan; 2 Department of Clinical Anatomy Development Studies, Gifu University Graduate School of Medicine, 1-1 Yanagido, Gifu City, 501-1194 Gifu Prefecture Japan

**Keywords:** Esophagectomy, Gastric conduit, Delayed gastric conduit emptying, Subtotal gastric conduit, Wide gastric conduit, Postoperative complications

## Abstract

**Purposes:**

Delayed gastric conduit emptying (DGCE) is a frequent complication after esophagectomy that affects nutrition and the quality of life. Subtotal gastric conduit (STG) and wide gastric conduit (WG) reconstructions are commonly performed. This study assessed whether WG reduces DGCE compared to STG.

**Methods:**

We retrospectively reviewed 169 patients who underwent posterior mediastinal gastric reconstruction between January 2019 and December 2024. Patients who underwent Ivor-Lewis procedures and non-gastric reconstructions were excluded. The conduits were classified as STG or WG based on the stapler firings. The resected gastric length and maximal diameter were measured. The outcomes included DGCE, anastomotic complications, pneumonia, weight loss, serum albumin, hemoglobin, and psoas muscle index (PMI). Appropriate statistical tests were performed.

**Results:**

Of the 169 patients, 128 underwent STG and 41 underwent WG reconstruction. The baseline characteristics were similar, although the WG group included more female patients and had a higher BMI. The resected gastric length was shorter in the STG (*p* < 0.001), whereas the conduit length tended to be longer (*p* = 0.051). No significant differences were observed in terms of DGCE, anastomotic leakage, pneumonia, weight loss, PMI, or albumin levels. Hemoglobin at 1 year was slightly lower in WG (10.2 vs. 11.1 g/dL, *p* = 0.002).

**Conclusions:**

WG reconstruction did not reduce DGCE or other complications compared with STG reconstruction. The gastric conduit size alone may not prevent DGCE. Further studies are warranted to explore surgical strategies for improving postoperative gastric emptying.

**Supplementary Information:**

The online version contains supplementary material available at https://doi.org/10.1007/s00595-026-03395-w.

## Introduction

 Esophageal reconstruction after esophagectomy is most commonly performed using a gastric conduit for reconstruction. Gastric conduit reconstruction is widely favored because it is technically simpler, requires fewer anastomoses, and is associated with a lower rate of postoperative complications than reconstruction using the small intestine or colon [1, 2]. Among the various gastric conduit techniques, subtotal gastric conduit (STG) reconstruction is one of the options. The advantages of STG include the preservation of intramural vascular networks, which ensures adequate blood flow at the anastomotic site (enhancing safety) and maintenance of gastric reservoir capacity (enhancing functionality) [3–7]. However, delayed gastric conduit emptying (DGCE) occurs relatively frequently [8–11]. DGCE can lead to thoracic stomach syndrome, manifesting as nausea, vomiting, early satiety, reflux esophagitis, and aspiration pneumonia, which may result in body weight loss and a reduced quality of life (QOL) [12–14]. At our institution, reconstruction is primarily performed using a wide gastric conduit (WG) in patients with abdominal esophageal or esophagogastric junction cancer based on oncological considerations. This study aimed to investigate whether WG can serve as an alternative to STG in preventing the development of DGCE.

## Materials & methods

### Study design and patients

**Fig. 1:**
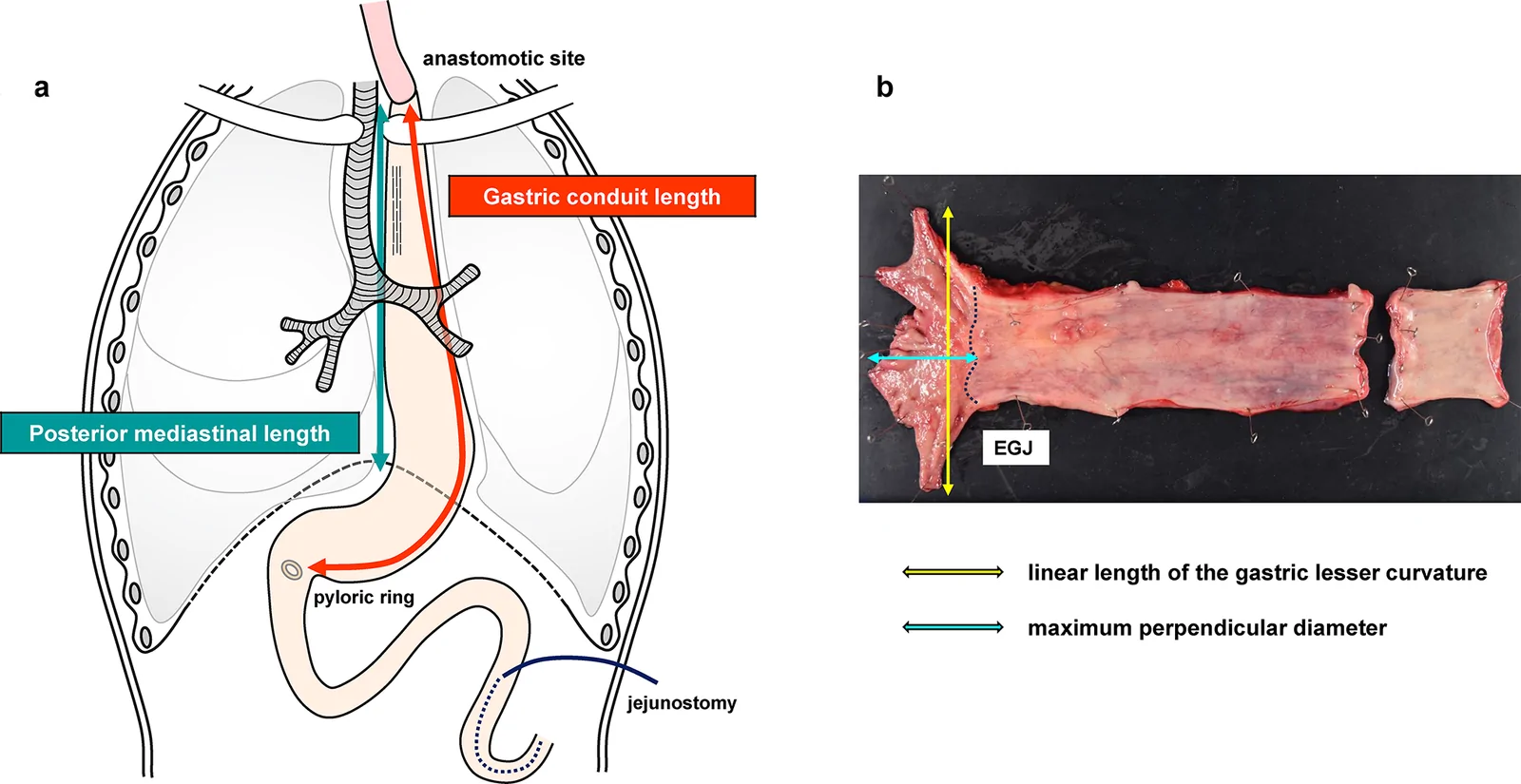
Flow of the patients included in the study. Of 191 esophagectomies performed, 169 cases were included in the analysis

This was a single-center, retrospective cohort study. The study population included patients who underwent esophagectomy at Gifu University Hospital between January 2019 and December 2024. All patients underwent reconstruction using our standard approach: a subtotal gastric conduit (STG) or wide gastric conduit (WG) elevated through the posterior mediastinal route with cervical hand-sewn anastomosis (Fig. 1).

### Exclusion criteria

The surgical exclusion criteria were as follows: Ivor Lewis reconstruction, retrosternal reconstruction, anterior mediastinal reconstruction, reconstruction using organs other than the stomach, and cases in which anastomosis was performed using methods other than hand-sewn techniques owing to tumor location. The postoperative exclusion criteria included patients who required reintubation or noninvasive positive-pressure ventilation after surgery.

### Selection and technique for gastric conduit construction

In all cases, the thoracic procedure was performed first under thoracoscopic or robot-assisted minimally invasive esophagectomy, followed by the abdominal procedure after patient repositioning. An esophageal surgeon performed laparoscopic perigastric lymphadenectomy and mobilization, followed by gastric transection through an upper midline laparotomy of approximately 7 cm in length. WG is primarily selected for patients with lower thoracic esophageal, abdominal esophageal, or gastroesophageal junction cancer to ensure an adequate proximal gastric margin. WG was also chosen for cases with gastric tumors, such as gastrointestinal stromal tumors located in the gastric fundus, or when intraoperative issues, such as bleeding during conduit creation, necessitated an extended resection.

**Fig. 2:**
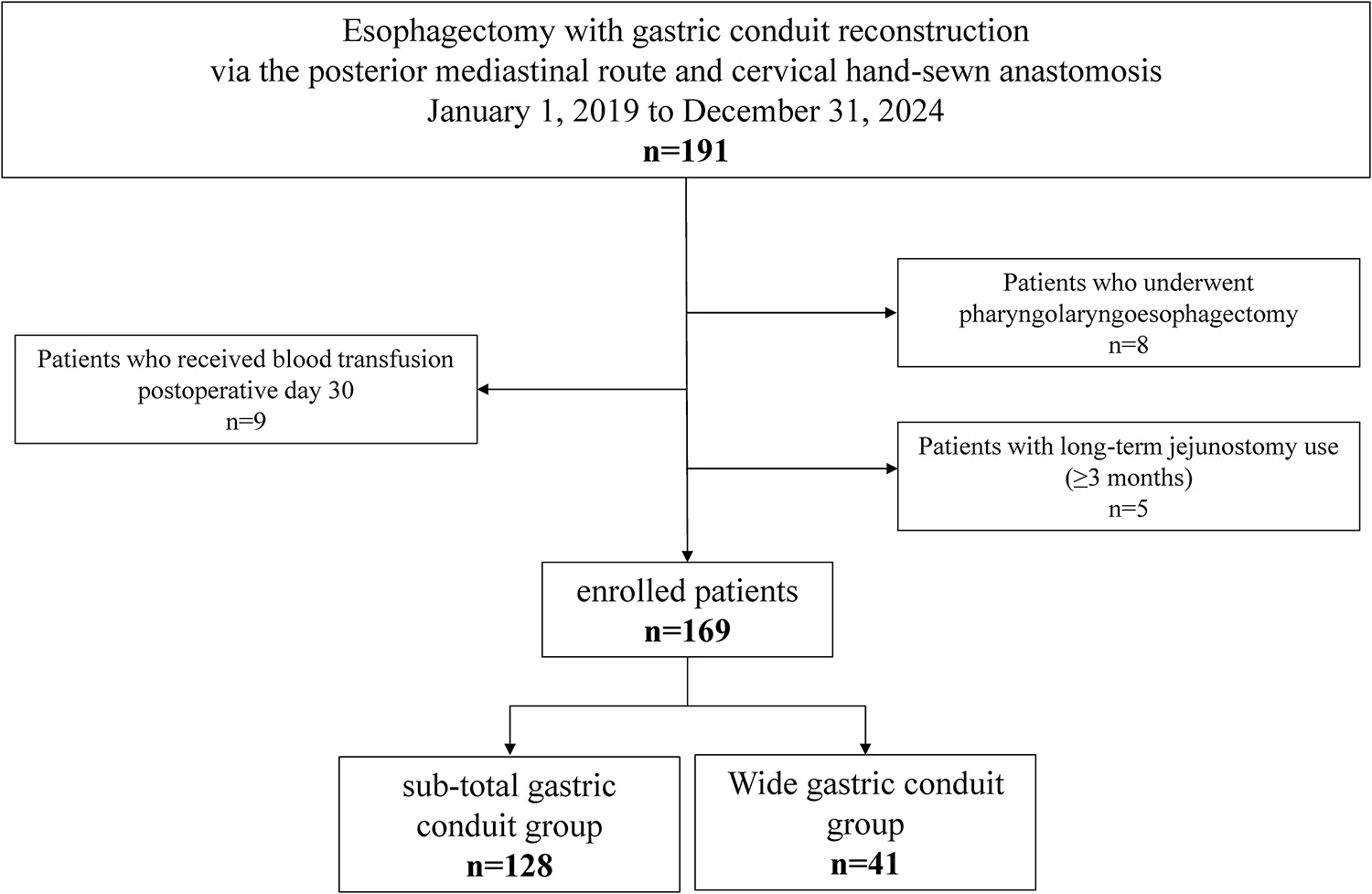
Creation of sub-total and wide gastric conduits using 60-mm staplers

In the WG group, a margin of 5 cm from the tumor edge was secured, and gastric transection was performed using three or more 60-mm linear staplers. In contrast, the STG group was constructed using one to two 60-mm linear staplers in all cases (Fig. 2).

### Measurement methods and diagnostic criteria

**Fig. 3:**
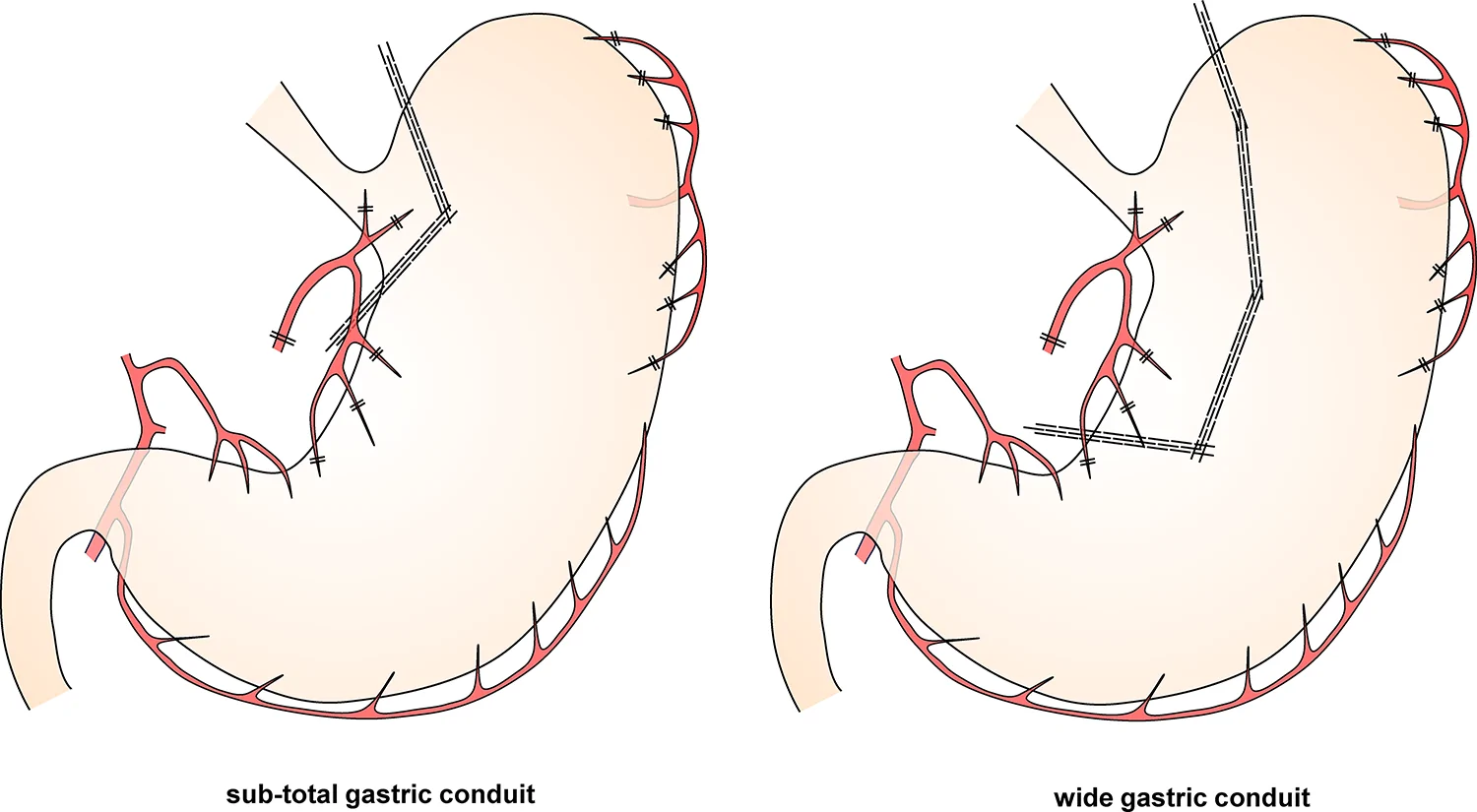
Measurement of the gastric conduit and posterior mediastinal route lengths. (a) Posterior mediastinal route length measured from the clavicle to the diaphragmatic crus; gastric conduit length measured along the greater curvature from the planned anastomotic site to the pylorus. Sub-total and wide gastric conduit lengths compared using the straight-line length along the lesser curvature and the maximal perpendicular width of the resected stomach

The length of the gastric conduit was measured intraoperatively from the planned anastomotic site along the greater curvature to the pyloric region. The length of the posterior mediastinal route was estimated using preoperative sagittal computed tomography (CT) images or by measuring the distance from the superior border of the clavicle to the diaphragmatic crura intraoperatively (Fig. 3a). To compare the sizes of the STG and WG, the linear distance along the lesser curvature of the resected specimenfrom the distal end to the esophagogastric junctionand the maximum diameter perpendicular to this line were measured (Fig. 3b).

Postoperative complications were defined as Clavien-Dindo grade ≥ II [15]. Anastomotic stricture was defined as the need for endoscopic balloon dilation. DGCE was diagnosed according to the criteria of the International Society for Diseases of the Esophagus [16], including clinical symptoms such as oral intake intolerance, early satiety, bloating, nausea/vomiting, and reflux, as well as radiological findings of gastric conduit dilatation and air-fluid levels on postoperative X-ray or computed tomography (CT). The imaging findings were independently assessed by at least two physicians. Early and late DGCE were classified according to a cutoff of 14 days post-surgery [16]. In all cases of DGCE, reinsertion of a gastric tube was performed, and endoscopic interventions or additional surgical procedures (e.g., pyloric dilation) were not required.

The psoas muscle mass was assessed using computed tomography images with the Synapse Vincent system (Fujifilm Co., Ltd., Tokyo, Japan). The cross-sectional area of the bilateral psoas muscles (cm²) was quantified automatically at the level of the third lumbar vertebra (L3). The psoas muscle index (PMI) was calculated by normalizing the total psoas muscle area at L3 (cm²) to the square of the patient’s height and expressed as cm²/m² [17–19]. All measurements were independently performed by two or more physicians, with no significant inter-observer differences observed.

### Surgical technique and postoperative management

The anastomotic site of the gastric conduit was set at the tip of the conduit in a maximally extended state on the greater curvature side of the anterior wall in all cases. After elevation through the posterior mediastinal route, hand-sewn anastomosis was performed [3], and the gastric conduit was subsequently secured to the diaphragmatic crura with as many sutures as feasible, up to a maximum of seven. Pyloroplasty was not performed in any case.

All patients were extubated on the morning following surgery and transitioned to oxygen supplementation via a facemask. In all cases, a nasogastric tube was placed across the anastomosis and routinely removed on postoperative day one. Postoperative analgesia and fluid management were conducted according to standardized protocols adjusted for patient body weight and hemodynamics, and no prokinetic agents were administered.

Jejunostomy was created in all patients at a site at least 35 cm distal to the ligament of Treitz, and enteral nutrition using an identical nutrient formulation was administered on postoperative day 2 in all patients according to a predetermined protocol for three months postoperatively, after which the jejunostomy was removed. In cases that developed DGCE, reinsertion of the gastric tube was performed, and endoscopic interventions or additional surgical procedures, such as pyloric dilation, were not required.

### Primary and secondary endpoints

The primary endpoints were the incidence of postoperative complications, including DGCE, anastomotic leakage, and pneumonia, as well as postoperative weight loss at 6 months and 1 year after surgery. The secondary endpoints were postoperative nutritional status, assessed by PMI and serum albumin, and hemoglobin levels as hematologic parameters for monitoring the overall patient condition.

### Statistical analysis

Continuous variables were assessed for normality using the Shapiro-Wilk test. Variables with a normal distribution are presented as the mean ± standard deviation (mean ± SD), whereas non-normally distributed variables are expressed as the median [interquartile range] (median [IQR]). Comparisons between groups were performed using the independent two-sample t-test for normally distributed variables and the Mann-Whitney U test for non-normally distributed variables. Categorical variables are presented as numbers (%), and group comparisons were conducted using the χ² test or Fisher’s exact test when any expected cell count was less than five. All statistical analyses were performed using R software (version 4.5.1) with the *tableone* package. Two-sided *p*-values < 0.05 were considered to be statistically significant.

## Result

After applying the exclusion criteria (Fig. 1), 169 patients were included, of whom 128 underwent STG reconstruction and 41 underwent WG reconstruction. The median age was 71 years, and 80% of the patients were male. Adenocarcinoma was present in 10 patients (5.9%), and 113 patients (66.9%) received neoadjuvant chemotherapy. Among all patients, eight had M1 disease. The median gastric conduit and posterior mediastinal route lengths were 34.0 cm and 25.9 cm, respectively. Postoperative complications included anastomotic leakage (1.2%), pneumonia (13.6%), and early DGCE (17.8%). The median length of hospital stay was 24 days, and the median postoperative weight loss was 7.57% at 6 months and 8.57% at 1 year (Table 1).

**Table 1:** Baseline patient characteristics and perioperative data

Characteristics	Data (*n*=169)
Age (median [IQR]), years	71.0 [63-77]
Sex (Male / Female), n (%)	135 (79.9) / 34 (20.1)
Body height (median [IQR]), cm	166.0 [159.8-170.6]
Preoperative weight (mean ± SD), kg	59.3 ± 11.1
Body mass index (mean ± SD), kg/m^2^	21.8 ± 3.33
Tumor location, n (%)	
Ce, Ut / Mt / Lt, Ae	35 (20.7) / 78 (46.2) / 56 (33.1)
Clinical T (UICC ver.8)	
1a / 1b / 2 / 3 / 4a / 4b	18 / 53 / 26 / 39 / 26 / 7
Clinical N (UICC ver.8)	
0 / 1 / 2 / 3	77 / 59 / 24 / 9
Clinical M (UICC ver.8)	
0 / 1	161 / 8
Clinical Stage (UICC ver.8)	
1 / 2 / 3 / 4a / 4b	59 / 27 / 38 / 39 / 6
Pathology (SCC / Adenocarcinoma), n (%)	159 (94.1) / 10 (5.9)
Neoadjuvant chemotherapy, n (%)	113 (66.9)
Gastric conduit length (median [IQR]), cm	34.0 [32.0-35.0]
Posterior mediastinal length (median [IQR]), cm	25.9 [23.8-26.6]
Gastric conduit – Mediastinum difference	
(mean ± SD), cm	8.25 ± 2.63
Anastomotic leakage, n (%)	2 (1.2)
Anastomotic stenosis, n (%)	19 (11.2)
Pneumonia, n (%)	23 (13.6)
Early DGCE, n (%)	30 (17.8)
Late DGCE, n (%)	14 (8.3)
Length of hospital stay (median [IQR]), days	24 [19–33]
Weight loss at 6 months (median [IQR]), %	7.57 [3.37–11.64]
Weight loss at 1 year (median [IQR]), %	8.57 [5.57–11.13]

Values are presented as mean ± standard deviation (SD) or median [interquartile range, IQR] as appropriate. Postoperative complications are classified according to Clavien-Dindo grade ≥ II. Ce, cervical; Ut, upper thoracic; Mt, middle thoracic; Lt, lower thoracic; Ae, abdominal esophagus; SCC, squamous cell carcinoma; DGCE delayed gastric conduit emptying

The patient characteristics according to the type of gastric conduit are summarized in Table 2. The median age was comparable between the STG and WG groups (71 vs. 69 years, *p* = 0.244). The proportion of male patients tended to be higher in the STG group than in the WG group (83.6% vs. 68.3%, *p* = 0.057). The median body height did not differ significantly between the groups (166 vs. 165.7 cm, *p* = 0.650). In contrast, preoperative weight and body mass index were significantly lower in the STG group than in the WG group (57.9 ± 11.2 vs. 63.7 ± 9.82 kg, *p* = 0.004; 21.1 ± 3.3 vs. 23.7 ± 2.4 kg/m², *p* < 0.001).

**Table 2:** Patient characteristics by type of gastric conduit

	STG group	WG group	*p*-Value
	*n*=128	*n*=41	
Age (median [IQR]), years	71 [62-77]	69 [63-73]	0.244
Male, n (%)	107 (83.6)	28 (68.3)	0.057
Female, n (%)	21 (16.4)	13 (31.7)	
Body height (median [IQR]), kg/m^2^	166 [160-170]	165.7 [158-171.8]	0.650
Preoperative weight (mean ± SD), kg	57.9 ± 11.2	63.7 ± 9.82	0.004*
Body mass index (mean ± SD), kg/m^2^	21.1 ± 3.3	23.7 ± 2.4	<0.001*
Tumor location (Ce, Ut / Mt / Lt, Ae)	31 / 69 / 28	4 / 9 / 28	<0.001*
Clinical stage (1/2/3/4a/4b) (UICC ver.8)	44 / 19 / 33 / 28 / 4	15 / 8 / 5 / 11 / 2	0.461
Neoadjuvant chemotherapy, n (%)	86 (67.2)	27 (65.9)	1.000
Pathology (SCC / Adenocarcinoma)	121 / 7	38 / 3	0.955
Preoperative psoas muscle index (median [IQR]), cm²/m²	5.83 [4.11-6.17]	5.92 [5.25-6.57]	0.414
Preoperative albumin (median [IQR]), g/dL	5.2 [4.4-5.5]	4.7 [3.8-5.1]	0.315
Preoperative hemoglobin (median [IQR]), g/dL	11.4 [9.9-12.1]	11.1 [9.6-11.5]	0.631
Resected gastric length (median [IQR]), cm	2.6 [2.4-2.9]	3.6 [3.4-4.3]	<0.001*
Maximum perpendicular diameter of resected specimen (median [IQR]), cm	4.7 [3.8-5.1]	6.9 [4.9-7.7]	<0.001*
Maximum width of the gastric conduit (median [IQR]), cm	8.1 [6.9-8.6]	7.1 [6.5-8.1]	0.211
Gastric conduit length (median [IQR]), cm	34 [32-35]	32 [31-35]	0.051
Posterior mediastinal length (median [IQR]), cm	26.4 [24.7-26.8]	25.5 [23.5-26.5]	0.049*
Gastric conduit – Mediastinum difference (mean ± SD), cm	8.50 ± 2.58	7.48 ± 2.67	0.031*

Values are presented as mean ± standard deviation (SD) or median [interquartile range, IQR] as appropriate

Ce, cervical; Ut, upper thoracic; Mt, middle thoracic; Lt, lower thoracic; Ae, abdominal esophagus; SCC, squamous cell carcinoma

Tumor location differed significantly between the groups (*p* < 0.001), whereas clinical stage, neoadjuvant chemotherapy administration, and histology were comparable between the groups. The resected gastric length was significantly shorter in the STG group than in the WG group (2.6 [2.4–2.9] cm vs. 3.6 [3.4–4.3] cm, *p* < 0.001). The gastric conduit length was slightly longer in the STG group, although the difference did not reach statistical significance (34 vs. 32 cm, *p* = 0.051). The posterior mediastinal route was marginally longer in the STG group than in the WG group (26.4 vs. 25.5 cm, *p* = 0.049), resulting in a greater mean gastric conduit–mediastinum difference (representing the intra-abdominal gastric conduit length) in the STG group (8.50 ± 2.58 vs. 7.48 ± 2.67 cm, *p* = 0.031).

**Fig. 4:**
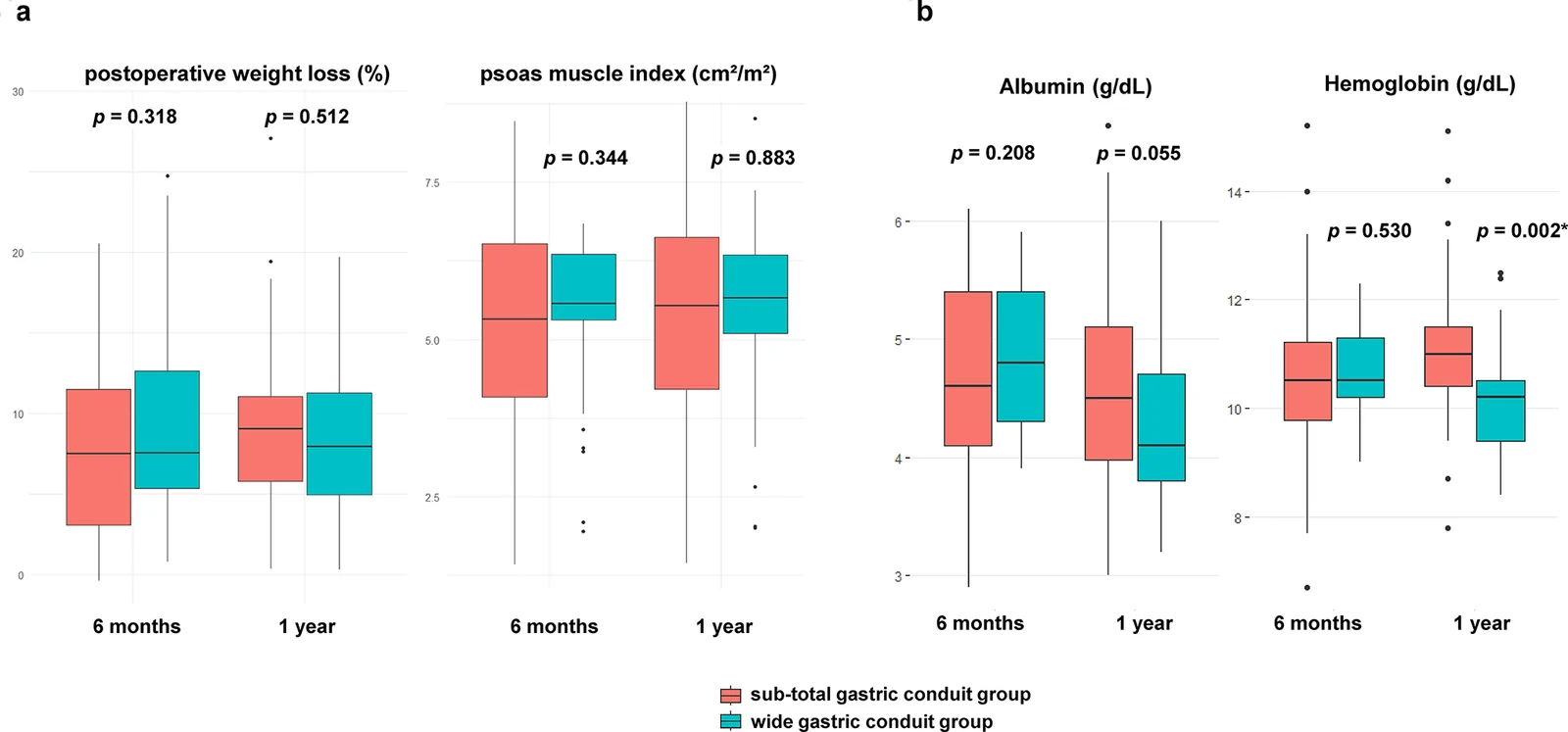
Postoperative functional outcomes in the two groups. (**a**) Body weight loss (%) and psoas muscle index at 6 months and 1 year. (**b**) Serum albumin and hemoglobin levels at 6 months and 1 year

The postoperative outcomes are presented in Table 3. There were no significant differences in the rates of anastomotic leakage, anastomotic stenosis, or pneumonia between the STG and WG groups, indicating comparable surgical safety. Similarly, there were no significant intergroup differences in the incidence of early or late DGCE, and no difference was observed in the duration of DGCE. The median length of hospital stay was comparable between the groups (24 vs. 23 days, *p* = 0.287). Postoperative weight loss at 6 months and 1 year did not differ significantly between the groups (*p* = 0.318 and *p* = 0.512, respectively) (Fig. 4a). In addition, no significant differences were observed when comparing PMI between the groups stratified by sex (Table 4). The serum albumin levels did not differ significantly at 6 months or 1 year. The hemoglobin levels were similar at 6 months. However, at 1 year, the hemoglobin levels were significantly lower in the WG group than in the STG group (11.1 vs. 10.2 g/dL, *p* = 0.002) (Fig. 4b).

**Table 3:** Postoperative data by type of gastric conduit

	STG group	WG group	*p*-Value
	*n*=128	*n*=41	
Anastomotic leakage, n (%)	2 (1.6)	0 (0)	0.568
Anastomotic stenosis, n (%)	16 (12.5)	3 (7.3)	0.529
Pneumonia, n (%)	17 (13.3)	6 (14.6)	1.000
Postoperative recurrence, n (%)	11 (8.6)	3 (7.3)	0.821
Early DGCE, n (%)	25 (19.5)	5 (12.2)	0.404
Late DGCE, n (%)	12 (9.4)	2 (4.9)	0.559
Duration of enteral nutrition (median [IQR]), days	91 [88-95]	93 [87-99]	0.911
Length of hospital stay (median [IQR]), days	24 [19-38]	23 [19-29]	0.287
Weight loss at 6 months (median [IQR]), %	7.5 [3.1-11.5]	7.6 [5.4-12.6]	0.318
Weight loss at 1 year (median [IQR]), %	9.0 [5.8-11.1]	8.0 [5.0-11.3]	0.512
Psoas muscle index at 6 months (median [IQR]), cm²/m²	5.32 [4.08-6.52]	5.56 [5.31-6.35]	0.344
Psoas muscle index at 1 year (median [IQR]), cm²/m²	5.47 [4.11-6.23]	5.51 [4.89-6.21]	0.883
Albumin at 6 months (median [IQR]), g/dL	4.9 [3.9-5.2]	4.8 [4.0-5.3]	0.208
Albumin at 1 year (median [IQR]), g/dL	5.1 [4.3-5.4]	4.1 [3.6-4.9]	0.055
Hemoglobin at 6 months (median [IQR]), g/dL	10.5 [9.8-11.3]	10.5 [9.8-11.3]	0.530
Hemoglobin at 1 year (median [IQR]), g/dL	11.1 [10.3-11.8]	10.2 [9.8-11.1]	0.002*
Serum iron at 6 months (median [IQR]), µg/dL	110 [66.4-118.5]	98 [58.4-105.5]	0.588
Serum iron at 1 year (median [IQR]), µg/dL	98 [58.7-98.8]	85 [45.8-97.9]	0.032*

Values are presented as median [interquartile range, IQR] as appropriate

Postoperative complications are classified according to Clavien-Dindo grade ≥ II

DGCE delayed gastric conduit emptying

**Table 4:** Comparison of psoas muscle index between two groups by sex

Male	STG group	WG group	*p*-Value
*n*=135	*n*=107	*n*=28	
PMI at 6 months (median [IQR]), cm²/m²	5.56 [4.67-6.65]	5.67 [5.50-6.37]	0.283
PMI at 1 year (median [IQR]), cm²/m²	5.81 [4.97-6.87]	6.04 [5.58-6.59]	0.597

**Female**	**STG group**	**WG group**	***p*** **-Value**
*n*=34	*n*=21	*n*=13	
PMI at 6 months (median [IQR]), cm²/m²	3.62 [2.85-4.13]	4.50 [3.28-5.31]	0.104
PMI at 1 year (median [IQR]), cm²/m²	3.62 [3.20-4.14]	4.21 [3.29-5.42]	0.096

Values are presented as median [interquartile range, IQR] as appropriate

PMI psoas muscle index

Subgroup analyses were performed using Firth’s penalized logistic regression to account for potential imbalances in baseline characteristics between the groups. The analyses were stratified by high BMI (≥ 25 kg/m²) and tumor location. No significant association was observed between the type of gastric conduit and DGCE in any of the subgroups (Supplementary Table 1).

## Discussion

This study aimed to evaluate whether WG can serve as a safe and effective alternative to STG, with a particular focus on its potential to reduce DGCE following esophagectomy. At our institution, STG is generally adopted as the standard reconstruction method. However, WG is selected in certain cases based on pre-defined oncological considerations. s. In the present study, all surgical procedures other than gastric conduit size were performed in a largely standardized manner, allowing us to evaluate the impact of conduit size on postoperative outcomes.

The STG and WG groups were classified according to the number of linear stapler firings used during gastric conduit construction. In addition, using fixed resected specimens, the length of the stomach along the lesser curvature and the maximum diameter perpendicular to this axis were measured to retrospectively assess the size and morphology of the gastric conduit used for reconstruction [20]. These analyses demonstrated that the STG was characterized by a longer conduit with a larger caliber than the WG.

The length of the posterior mediastinal route was significantly shorter in the WG group, which may be attributable to the higher proportion of female patients in this group. Furthermore, patients in the WG group had a higher body mass index. This finding may reflect the increasing number of patients with esophagogastric junction cancer associated with obesity and gastroesophageal reflux disease included in the WG group.

The outcomes between the groups were evaluated from both safety and functional perspectives. No significant differences were observed in the postoperative complications, including DGCE. Similarly, postoperative weight loss at 6 months and 1 year did not differ significantly between the groups. Previous studies have shown that DGCE occurs more frequently in female patients as a patient-related factor and in cases with greater differences between the gastric conduit length and mediastinal route length as a surgery-related factor. Risk factor analyses have also suggested that female sex and a higher BMI may contribute to the development of DGCE [21]. In the present study, although the WG group tended to include a higher proportion of female patients and had a significantly higher BMI, no significant differences in safety outcomes were observed compared with those in the STG group. Logistic regression analyses were performed to identify the risk factors for the development of DGCE (Table 5). In the multivariate analysis, the type of gastric conduit was not significantly associated with DGCE, and no independent risk factors were identified. Further investigation is warranted regarding the association between the development of DGCE and the discrepancy between gastric conduit length and mediastinal route length (size mismatch), as well as the presence of preexisting pulmonary comorbidities. Skeletal muscle mass, as assessed by PMI, a surrogate marker for sarcopenia, did not differ significantly between the two groups, consistent with serum albumin levels. PMI was analyzed separately by sex, and no significant differences were observed between the groups at either 6 months or 1 year postoperatively. In contrast, the postoperative hemoglobin levels appeared to be slightly lower in the WG group.

**Table 5:** Factors associated with early DGCE: Univariable and Multivariable Firth Logistic Regression

	Univariable OR (95% CI)	*p*-Value	Multivariable OR (95% CI)	*p*-Value
Age (per 1 year increase)	0.97 (0.93–1.01)	0.12	0.96 (0.92–1.00)	0.31
Sex (male = reference)	0.56 (0.16–1.57)	0.31	0.49 (0.12–1.595)	0.27
Body mass index (per 1 kg/m² increase)^)^	1.05 (0.93–1.18)	0.43	1.09 (0.94–1.25)	0.25
Posterior mediastinal length (per cm)	0.95 (0.80–1.12)	0.51	0.87 (0.72–1.06)	0.16
Gastric conduit length (per cm)	1.11 (0.93–1.14)	0.21	1.12 (0.90–1.41)	0.32
Gastric conduit – Mediastinum difference (per cm)	1.13 (0.97–1.33)	0.13	0.72 (0.24–2.2)	0.62
Gastric conduit type (WG vs. STG)	0.57 (0.18–1.5)	0.29	0.52 (0.16–1.52)	0.26

OR: odds ratio; 95% CI: 95% confidence interval; WG: wide gastric conduit; STG: subtotal gastric conduit

OR represents the odds of DGCE relative to the reference for categorical variables or per unit increase for continuous variables (mediastinal and conduit lengths per 1 cm, conduit–mediastinum difference per 1 cm). Male is the reference for sex. Firth’s logistic regression was used to reduce bias from limited events. *p* < 0.05 considered significant

It is well established that patients undergoing esophagectomy often develop anemia due to iron and trace elements [22–24]. Similarly, anemia is known to persist following bariatric surgery, such as sleeve gastrectomy, which involves tubular resection of the stomach [25, 26]. Multiple studies have also demonstrated that the extent of gastric resection is associated with the frequency of postoperative anemia [20, 27–30]. In the present study, there were differences in the extent of gastric resection between the STG and WG groups, and a significant difference was also observed in the postoperative serum iron levels between the two groups. These findings may have influenced the incidence of postoperative anemia. However, the possibility that postoperative anemia in the WG group was influenced by the higher proportion of female patients cannot be excluded. Furthermore, the evaluation of ferritin levels and vitamins remains an important issue for future investigations.

Surgical modifications have also been investigated as strategies for preventing DGCE [31, 32]. Yano et al. reported pilot studies in which the duodenum was transected during esophagectomy, and prophylactic Roux-en-Y anastomosis was added to prevent DGCE [33]. This approach was subsequently evaluated in a randomized controlled trial, which demonstrated that the Roux-en-Y group experienced significantly reduced bile reflux and improved postoperative QOL [34]. Additionally, Fujimoto et al. performed a double-tract reconstruction in which the gastric conduit was anastomosed side-to-side with the elevated jejunum to create a “second outlet” [35]. This modification was reported to enhance gastric conduit emptying, and endoscopic examinations showed no evidence of retained food or reflux esophagitis. Moreover, the ATHLETE trial, led by Kitadani et al., is currently underway as a randomized controlled trial with the primary endpoint of anastomotic leakage, aiming to evaluate the potential superiority of STG over WG or reconstruction using a narrow-diameter gastric tube [36]. Incorporating such surgical techniques may provide a novel approach for addressing DGCE. Furthermore, the impact of the extent and transection line of gastric resection during gastric conduit creation on the regulatory mechanisms of gastrointestinal motility associated with the gastric pacemaker remains an important issue for future investigation.

This study has several limitations. First, it was a single-center, retrospective investigation. Second, differences existed in the oncological background, particularly between adenocarcinoma and squamous cell carcinoma. Third, no standardized criteria were applied regarding the administration of iron or vitamin supplements or the specific duration of enteral feeding via jejunostomy.

## Conclusion

WG did not demonstrate a significant reduction in postoperative complications, including DGCE, compared to STG. The introduction of novel surgical techniques, such as double-tract reconstruction, therefore warrants further evaluation in multicenter studies.

## Electronic Supplementary Material

Below is the link to the electronic supplementary material.

Supplementary material 1

## Data Availability

The datasets generated and analyzed during the current study are available from the corresponding author upon reasonable request.
