# Supplementary material for: Conduit size and the risk of delayed gastric conduit emptying after esophagectomy
Source: Surg Today. 2026 Jul 14;56(11):2708–16. doi: 10.1007/s00595-026-03395-w (PMC13722765; doi:10.1007/s00595-026-03395-w)
Supplement: Supplementary file 1 [file 595_2026_3395_MOESM1_ESM.docx]

| **Supplementary Table 1.** Subgroup analysis for DGCE (Firth penalized logistic regression) | | | | |
| --- | --- | --- | --- | --- |
| **subgroup** | **variable** | **OR** | **95% CI** | ***p*-value** |
| high BMI (>25) | STG vs WG | 1.36 | 0.08-23.6 | 0.803 |
| high BMI | Sex |  |  | 0.683 |
| high BMI | Lt, Ae vs Ce, Ut |  |  | 0.102 |
| high BMI | Lt, Ae vs Mt |  |  | 0.544 |
| Lt, Ae | STG vs WG | 0.74 | 0.12-4.60 | 0.738 |
| Lt, Ae | Sex |  |  | 0.354 |
| Lt, Ae | BMI |  |  | 0.758 |
| Ce, cervical; Ut, upper thoracic; Mt, middle thoracic; Lt, lower thoracic; Ae; BMI, body mass index; DGCE, delayed gastric conduit emptying. | | | | |
